# Spatial pattern and determinants of institutional delivery in Ethiopia: Spatial and multilevel analysis using 2019 Ethiopian demographic and health survey

**DOI:** 10.1371/journal.pone.0279167

**Published:** 2023-02-16

**Authors:** Mukemil Awol, Dejene Edosa, Kemal Jemal

**Affiliations:** 1 Department of Midwifery, College of Health Sciences, Salale University, Fitche, Ethiopia; 2 Department of Nursing, College of Health Sciences, Salale University, Fitche, Ethiopia; Baldwin Wallace University, UNITED STATES

## Abstract

**Background:**

In Ethiopia, despite the progress that has been made to improve maternal and child health, the proportion of births occurring at health institutions is still very low (26%), Which significantly contribute to a large number of maternal death 412 deaths/100,000 live births. Therefore, this study intended to determine spatial pattern and factors affecting institutional delivery among women who had live birth in Ethiopia within five years preceding survey.

**Method:**

Data from 2019 Ethiopian demographic and health survey were used. Taking into account the nested structure of the data, multilevel logistic regression analysis has been employed to a nationally representative sample of 5753 women nested with in 305 communities/clusters.

**Result:**

A significant heterogeneity was observed between clusters for institutional delivery which explains about 57% of the total variation. Individual-level variables: primary education (OR = 1.8: 95% CI: 1.44–2.26), secondary education (OR = 3.65: 95% CI: 2.19–6.1), diploma and higher (OR = 2.74: 95% CI: 1.02–7.34), women who had both Radio and Television were 4.6 times (OR = 4.6; 95% CI: 2.52, 8.45), four and above Antenatal visit (AOR = 2.72, 95% CI:2.2, 3.34), rich wealth index (OR = 2.22; 95% CI: 1.62–2.99), birth interval for 18 to 33 months (OR = 1.8; 95% CI: 1.19, 2.92), and women who space birth for 33 and above months (OR = 2.02; 95% CI: 1.3, 3.12) were associated with institutional delivery. Community level variables, community high proportion of antenatal visit (OR = 4.68; 95% CI: 4.13–5.30), and Region were associated with institutional delivery.

**Conclusion:**

A clustered pattern of areas with low institutional delivery was observed in Ethiopia. Both individual and community level factors found significantly associated with institutional delivery theses showed the need for community women education through health extension programs and community health workers. And the effort to promote institutional delivery should pay special attention to antenatal care, less educated women and interventions considering awareness, access, and availability of the services are vital for regions. A preprint has previously been published.

## Background

Childbirth is a complex process, and it is essential to remember to provide everything that is needed to ensure both the mother and newborn child receive the safest care possible [1]. Home birth is most common among the poor. In sub-Sahara Africa (SSA), South Asia, and Southeast Asia, 74.7–89.9% of women in the lowest two wealth quintiles reported giving birth at home [2–4]. As stated in the Ethiopian demographic health survey (EDHS) 2016 report, about 74% of births took place at home without skilled attendants [5]. In ideal circumstances, women giving birth in facilities rather than at home could avoid up to 14 perinatal deaths per 1000 births [6].

The World Health Organization (WHO) estimates up to 15 percent of expected births worldwide develop life-threatening complications during pregnancy, delivery or the postpartum period [7].Almost all maternal deaths (99%) occur in developing countries. And over half of these fatalities take place in Sub-Saharan Africa, with nearly one-third occurring in South Asia [11].

In Ethiopia Based on national Maternal Death Surveillance and response (MDSR) annual reports in 2015/16 about 633 mothers were died due to maternal causes [8]. In 2015, approximately 8700 kids were born every day, with nearly 240 of these newborns dying before attaining their first month. Institutional delivery still very low (only 26%), this worsen maternal and neonatal deaths during the first 24 hours after delivery [7, 9].

Pregnancy and postpartum health care are essential for both the mother’s sustenance and the infant’s well-being. Prenatal care that is skilled Childbirth and the postpartum are critical measures for lowering mothers and newborns illness and death [10]. As a result, almost all of the maternal deaths that occur every day in developing countries could have been avoided if comprehensive health care services had been available [9].

Over the last couple of decades, the Ethiopian government has stepped up efforts to develop pro-poor policies and strategies in order to readjust health care services toward health promotion, disease prevention, and curative services. The Health Extension Program (HEP), the country’s flagship program, provides low-cost essential services to all Ethiopians, primarily women and children [11]. In response to health worker shortages and unequal distribution, the Ethiopian government has successfully scaled up the health manpower via incredibly rapid midwifery training, Integrated Emergency Surgery and Obstetrics (IESO), and community-based services. This resulted in a four to five fold increase in human resources and facilities in less than a decade [11, 12].

The supply-side policy changes markedly increased the accessibility and geographical availability of healthcare centers. As a consequence of these policies and interventions, over 90 percent of the rural population has access to a healthcare facility within a two-hour walk, and 62 percent of women who had a live birth received Antenatal car (ANC) from a skilled provider at least once for their most recent birth [10, 13, 14].

Due to the obvious efforts made thus far, the government of Ethiopia made a significant 39 percent reduction in maternal mortality ratio from 2011 to 2016, and in order to maintain the momentum, the ministry of health developed and endorsed a national reproductive health strategy for the years 2016–2020. This strategy reaffirms the ministry’s commitment by establishing targeted and measurable agendas to improve women’s and children’s health [12]. Despite this massive effort to increase community utilization of health services, institutional childbirth and skilled delivery attendance remains low [10].

Home delivery is still widely practiced across the country. On the other hand, the country must work hard to meet the ambitious Sustainable Development Goal (SDG) 2030 target of 70/100,000 maternal deaths and the Health Sector Transformation Plan (HSTP) target of 199/100,000 live births MMR in 2020 [10, 12, 15].

As a result, there is a significant gap between what was planned and what was accomplished.

In addition, the country is on track to meet the Sustainable Development Goal (SDG) target of 2030.Various studies are needed to support this ambitious plan established by the Sustainable Development Goal.

Many studies have sought to identify factors associated with place of delivery over the years, with many studies corroborated in reporting significant associations with maternal education, household wealth, maternal age, health facility distance, or lack of transportation [16–18]. However, there is a gap in determining the impact of higher level factors and geographical variation of institutional delivery. Furthermore, in countries such as Ethiopia, where resources are scarce, there were few opportunities to collect country-level data; however, analyzing data from EDHS enabled us to provide country-level figures. It is also critical to identify modified and persistent factors that contribute to the failure of future health plans. To fill this gap, we conducted spatial pattern, individual and community-level factors associated with institutional delivery in Ethiopia: using data from the 2019 Ethiopian Demographic and Health Survey.

## Methods and materials

Ethiopia is located in the Horn of Africa and shares a border with Eritrea, Djibouti, Somalia, Sudan, South Sudan, and Kenya. The country covers an area of 1.1 million km2 (square kilometer) with geographical diversity, ranging from 4,550 meters (m) above sea level down to the Afar depression 110m below sea level, which is comprised of over 80 ethnicities and speaking over 80 different languages [19]. Administratively, Ethiopia is divided into nine regional states and two city administrations subdivided into 68 zones, 817 districts, and 16,253 kebeles (lowest local administrative units of the country) in the administrative structure of the country [20]. Based on the 2018 world bank report Ethiopia had a total population of 109 million with a gross national income per capital of US$ 790 [21]. Ethiopia’s health system comprises three tiers: a primary health care unit, a general hospital, and a specialized hospital [22, 23].

### Source of data

The data came from EDHS 2019, specifically the under-five children’s file (KR) (http://www.measuredhs.com). We were able to download the datasets after the measurement program allowed us to do so. The unweighted sample consisted of 5753 women who had live births in the five years preceding the survey. The 2019 EDHS sample was stratified and selected in two stages, and interviews were conducted face-to-face with permanent residents and visitors who stayed in the residences the day before the survey. The 2019 EDHS sampling frame is a composite of all census enumeration areas (EAs) created for the upcoming 2019 Ethiopia Population and Housing Census (PHC) conducted by the Central Statistical Agency (CSA). The census frame includes the complete list of 149,093 EAs created for the 2019 PHC. An EA is a geographical area with an average of 131 households. The sampling frame includes data on the EA’s location, type of residence (urban or rural), and the estimated number of residential households [24]

### Study variables

The outcome variable for this study was institutional delivery, which was coded as “0” if the women gave birth at home and “1” if the women gave birth at a health facility. Institutions/facilities delivery was stated as the births at health institution/facility within five years afore the survey.

### Independent variable

**Individual level factors** were women education level, household wealth index, birth interval, number of antenatal care visit, age of the women, media exposure and marital status.

**Community level factors** were residence, region, community educational status, community ANC coverage and community poverty. The EDHS did not collect data that can directly describe the clusters’ characteristics except the place of residence and region. Therefore, other common community-level data were generated by aggregating the individual characteristics with our interest in a cluster. The aggregates were computed using the proportion of a given variables’ subcategory we were concerned on in a given cluster. Since the aggregate value for all generated variable was not normally distributed. It was categorized into groups based on the national median values.

### Operational definition

#### Exposure to mass media

A frequency of listening to the radio and watching television were considered exposure to mass media in this study by excluding exposure to magazines and newspapers. So, women exposed to either television or radio at least once per week considered exposed, if not exposed at all, taken as not exposed [20].

#### Community women education

Was defined as the proportion of mother’s who attended primary/secondary/ higher education within the cluster. The aggregate of individual mother’s primary/secondary/higher educational attainment can show the overall educational status of women within the cluster. There were two categories for this variable with reference to the national median value: higher proportion of mother’s who attended primary/secondary/higher education and lower proportion of mother’s who attended primary/secondary/higher education within the cluster.

#### Antenatal care utilization

Was defined as mothers who had at least four antenatal care visit [25].

#### Community antenatal coverage

The proportion of women in the clusters who had four and above antenatal care (ANC) from a skilled provider during the pregnancy of last delivery.

#### Community poverty status

It is defined as the proportion of poor or poorest mothers within the cluster. Within the cluster proportion of poor or poorest were aggregated and show over all poverty status within the cluster.

### Data management and statistical analysis

The 2019 EDHS data were pre-tested before the actual data collection. Data collectors had received training in interviewing techniques, field procedures, the content of the questionnaires, and how to administer both paper and electronic questionnaires; after all, questionnaires were finalized in English, then translated into Amarigna, Tigrigna, and Oromiffa [24]. Since this was secondary data, the data were maintained by processing, editing, raw coding data, and re-coding, checking its completeness, and cleaning the missing values by running frequencies based on the research’s interest. Sample weights were applied to compensate for the disproportional probability of sampling and non-response rate between the strata that have been geographically defined. A detailed explanation of the weighting procedure can be found in the EDHS final report [24]. Cross tabulations and summary statistics were used to describe the study population.

### Spatial analysis

The aggregated home and health facility delivery count data were joined to the geographic coordinates based on each cluster unique identification code. Global spatial autocorrelations were assessed with ArcGIS version 10.5 using the Global Moran’s I statistic (Moran’s I) to evaluate whether the pattern expressed is clustered, dispersed, or random across the study areas. Moran’s I values close to −1 indicated institutional delivery were dispersed, whereas I values close to +1 indicated institutional delivery were clustered, and distributed randomly if I value was zero. A statistically significant Moran’s I (p < 0.05) led to the rejection of the null hypothesis, and indicated the presence of spatial autocorrelation as well as it detect the existence of at least one cluster, but not the specific location of the cluster(s) [26].

For positive global spatial autocorrelation, local spatial association indicators were used to assess clusters and outliers by comparing the values in each specific location with values in neighboring locations. It allows for decomposing the pattern of spatial association into four categories (quadrants) called Hot spot analysis [27]. And this help us to identify the proportion of institutional delivery based on sampled enumeration area. Since geographic coordinates were collected at the cluster level, the unit of spatial analyses was 2019 EDHS clusters. Finally, we employed Kulldorff’s purely spatial scan statistic method using the Bernoulli probability model in SaTScan version 9.6 software to detect the local spatial clusters of areas with high home delivery. Its output presents the hotspot areas in circular windows, indicating the areas inside the windows are higher than expected distributions compared to the areas outside of the cluster windows [28]. We used a maximum 50% of the popul30+ation at risk for the spatial cluster size. A cluster was statistically significant if a p-value < 0.05. Interpolation- we run the empirical Kriging technique to predict values for areas where data points were not taken.

### Multilevel analysis

First a descriptive analysis was conducted for all individual- and community-level variables in order to examine the characteristics of the sample. Considering this hierarchical nature of the data and the assumption of independence among individuals within the same community and the assumption of equal variance across the community is violated in nested data. Therefore, flat models could underestimate the effect sizes’ standard errors and lead to bias (loss of power or type I error), affecting the null hypothesis [29]. Hence, in order to account the hierarchical nature of the EDHS data and response variable multilevel logistic regression analysis was implemented to test the effect sizes of individual and community level factors on women’s decision to place of delivery. During analysis, the characteristics of women were taken as individual level (level-1) and characteristics of clusters were treated as community level (level-2).

Model I (Empty model) was fitted without explanatory variables to test random variability in the intercept and to estimate the intra class correlation coefficient (ICC).

$$\text{The}\;\text{intra-class}\;\text{correlation}\;\left(\text{rho}\right)\;\rho =\frac{\sigma 2\text{uo}}{\frac{\pi 2}{3}+\sigma 2\text{uo}}$$

Where σ^2^_uo_ = variance due to group level error term (u_oj_) and π^2^/3 is level-1 variance.

Model II examined the effects of individual level characteristics, Model III examined the effect of community level variables and Model IV examined the effects of both individual and community level characteristics simultaneously. The p value <0.05 was considered as statistically significant. For measurements of variation (random effects), intra-class correlation coefficient (ICC), median odds ratio (MOR), and proportional change in variance (PCV) statistics were computed. Model comparison was made based on Akakie Information Criteria (AIC) and Deviance Information Criteria (DIC). The model with the lowest information criterion was considered to be the best fit model [29].

### Ethical considerations

For this study, The ethical clearance was obtained from Salale University ethics Committee.The data were obtained and used with the Central Statistical Agency of Ethiopia’s prior permission. We registered for dataset access and wrote the study’s title and significance on the website after completing a short registration form. Downloading of datasets was done using the accessed website at http://www.measuredhs.com on request with the help of ICF international. Downloading data were used only for this study. The dataset was not passed on to other researchers without the consent of EDHS. All EDHS data were treated as confidential, no need to identify any household or individual respondent interviewed in the survey.

## Results

### Description of individual level and community level characteristics

This study focused on a sample of 5753 unweighted data of women and 5526.9365 weighted women who had live births in the last five years preceding EDHS 2019 survey. More than half of the women practice home delivery (52.46%). the mean age of respondents was 28.6 ± 6.5 and most of them were in the age group of the 20-29years (50.4%) and 30–40 years (36%). A large proportion of women had birth interval above 33 months (54%) of them 28.22% gave birth at home. Only 8.94% of the women space their children less than 18 months from these 6.13% gave birth at home and 36.96% space between 18 to 33 month out of these 24.21% gave birth at home.

Nearly half (43%) of individual had four and above ANC visit out of them 11.33% gave birth at home on the other hand 57% of respondent ranged between one to three ANC visit of them 37.11% gave birth at home. Similarly, the household’s cumulative living standard was expressed by three quintiles. As such, the proportion of women was nearly equaled among the wealth quintiles from (35.55%) in richest out of these 26.3% participant gave birth at health facility to 45.56% in the poorest with majority (32.5%) delivered at home. the other 18.88% were classified under middle class.

Concerning community level variable one third (75%) of participant were from rural out of them 45% of gave birth at home and 25% were from urban residence with 52% health facility delivery. About 55% of community had high proportion of education out of this only 31.16% gave birth at health facility (for detail see Table 1).

**Table 1:** Individual and community level characteristics of women who had live births in the last five years preceding (EDHS) 2019, Ethiopia.

Individual level variable	N (%)	N (%)	Proportion of place of delivery	
	un-weighted	weighted		
			Home delivery	Health facility
**Marital status**				
Never married	156 (2.71%)	126.99879 (2.30%)	61.3 (1.11%)	65.67(1.2%)
Married	5,355 (93.08%)	5188.554 (93.88%)	2724(49.3%)	2,465 (45%)
Widowed	61 (1.06%)	65.66 (1.19%)	46.6(0.84%)	19(0.34%)
Divorced	181 (3.15%)	145.72671 (2.64%)	67.4(1.22%)	78.3(1.42%)
**Women Educational level**				
No education	3,149 (54.74%)	2961.606(53.6%)	1992.3(36.05%)	969.3(17.54%)
Primary	1,82(31.69%)	1956.4365 (35.4%)	826(14.95%)	1,130.3(20.5%)
Secondary	480 (8.34%)	414.93275 (7.51%)	67.4(1.22%)	347.5 (6.29%)
Higher	301 (5.23%)	193.96084 (3.51%)	13.4(0.24%)	180.5(3.27%)
**Media Exposure**				
Not at all	3,755 (65.27%)	3638.174(65.83%)	2,276.9(41.2%)	1,361.3(24.6%)
Either radio or television	1,459 (25.36%)	1491.319(26.98%)	579.5 (10.5%)	911.8 (16.5%)
Both radio and television	539 (9.37%)	397.4446 (7.19%)	42.93(0.78%)	354.5(6.41%)
**Community level variable**				
**Community women education**				
Low	2,274 (39.53%)	2464.028(44.58%)	1,858.93(33.6%)	905.4(16%)
High	3,479 (60.47%)	3062.909(55.42%)	1,040.4(18%)	1722.2(31.16%)
**Community poverty**				
Low	2,275 (39.54%)	2461.66(44.54%)	1,040.4(16.4%)	1,556.8(28.2%)
High	3,478 (60.46%)	3065.27(55.46%)	1,994.5(36%)	1,070.8(19.4%)
**Community ANC coverage**				
Low	3,391(44.7%)	2087.512(37.77)%	2,270.4(41%)	888.7(16.1%)
High	1,675(22.07%)	3439.424(62.23%)	628.95(11.4%)	1,738.9(31.5%)
**Residence**				
Urban	1,328 (23.08%)	1366.9 (24.73%)	404.78(7.3%)	962.1(17.4%)
Rural	4,425 (76.92%)	4160.035(75.27%)	2,494.55(45.2%)	1,665.4(30.1%)
**Region**				
Tigray	454 (7.89%)	371.3063 (6.72%)	102.5(1.8%)	268.8(4.9%)
Afar	652 (11.33%)	85.94(1.55%)	61.6(1.1%)	24.29(0.4%)
Amhara	511 (8.88%)	1049.9(19.00%)	480.85(8.7%)	569.02 (10.3%)
Oromia	719 (12.50%)	2210.99(40.00%)	1,305.3(23.6%)	905.63 (16.4%)
Somali	637 (11.07%)	408.51(7.39%)	313.4 (5.7%)	95.07 (1.7%)
Benishangul	530 (9.21%)	67.26(1.22%)	24.4 (0.4%)	42.8 (0.78%)
SNNPR	660 (11.47%)	1105.92(20.01%)	580.6(10.5%)	525.3 (9.5%)
Gambela	450 (7.82%)	24.702(0.45%)	7.3 (0.13%)	17.35 (0.31%)
Harari	447 (7.77%)	16.4 (0.30%)	5.9 (0.11%)	10.46 (0.2%)
Addis Ababa	291 (5.06%)	156.213(2.83%)	8.05(0.15%)	148.17(2.7%)
Dire dawa	402 (6.99%)	298.4(0.54%)	9.2(0.2%)	20.6 4(0.34%)

### Result of spatial analysis

#### The spatial pattern of institutional delivery

**Fig 1:**
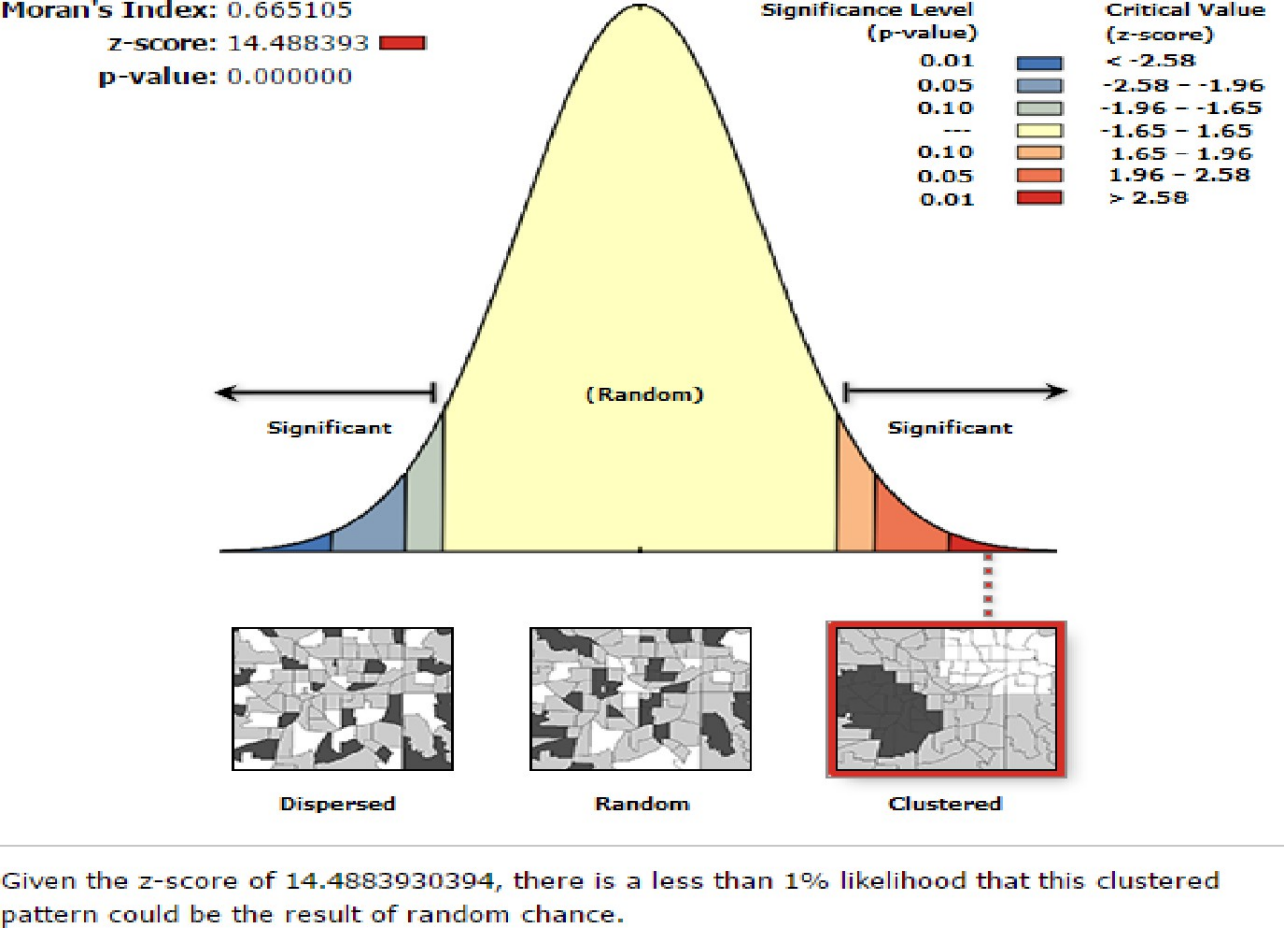
Global spatial autocorrelation of institutional delivery based on feature locations and attributes values across the study areas among women who had live birth in the last five years preceding 2019 EDHS.

The global spatial autocorrelation analysis based on feature locations and attribute values show that, the pattern of institutional delivery was clustered across Ethiopia. (Global Moran’s I = 0.665105, p-value < 0.000) **(see Fig 1**).

#### Spatial distribution of institutional delivery

**Fig 2:**
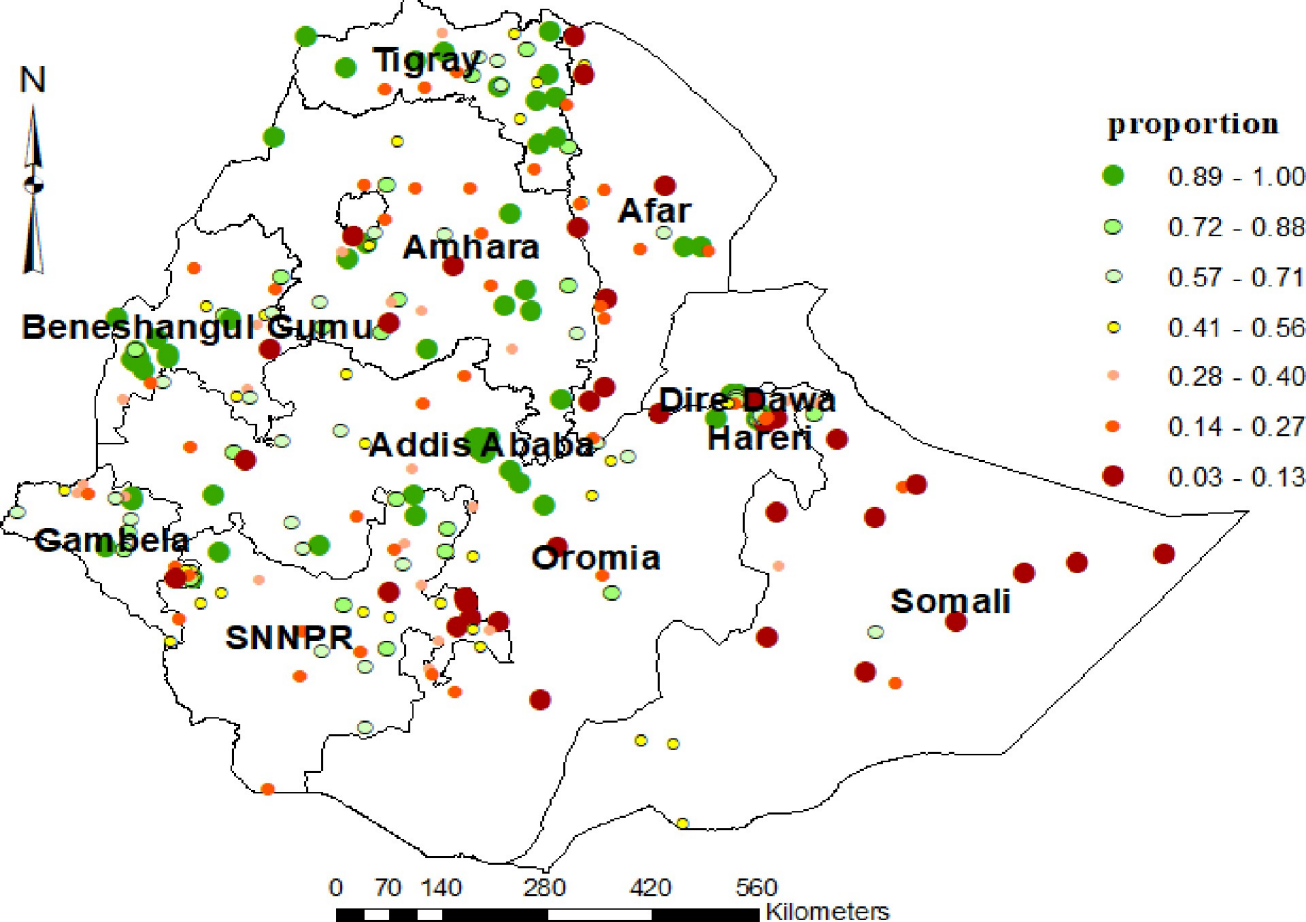
Hot spots analysis of institutional delivery among women who had live birth in the last five years preceding 2019 EDHS.

As evidenced via hot spot analysis the proportion of institutional delivery remains markedly low, which ranged between (3% to13%) mostly in the Somali region followed by Afar region, Harari, part of SNNPR, and some part of (Amhara region and Oromia regions). Conversely, high proportion of institutional delivery were located in Addis Ababa, Tigray, Dire Dawa, Gambela and Benishangul-Gumuz. The circle’s color and size indicate the proportion of delivery to either side. This means that as the size and intensity of the colour increase, so does the proportion.(for detail **see Fig 2**).

### Interpolation result

We estimated the spatial distribution of institutional delivery for areas where data points were not taken across Ethiopia by using empirical Kriging technique. Measured the distance from the known point to predict unknown points/areas and indicated the point in the ranges of the event occurrences.

**Fig 3:**
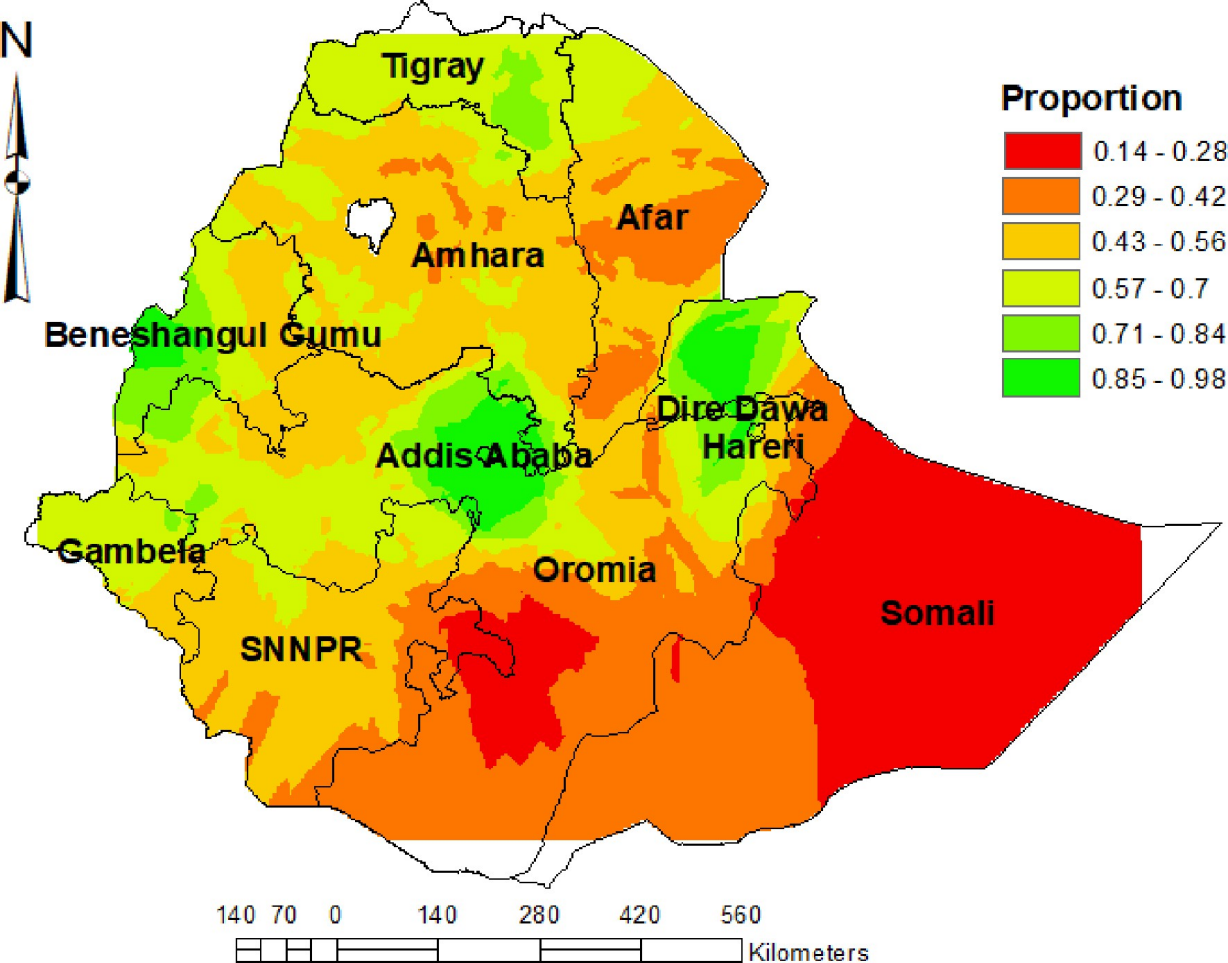
Interpolation of institutional delivery among women who had live birth in the last five years preceding 2019 EDHS.

More than 85% of women who live in an area with red color had institutional delivery. Whereas less than 14% of women who resided in an area with green color have had institutional delivery (for detail **see Fig 3**).

### SaTScan spatial analysis

**Fig 4:**
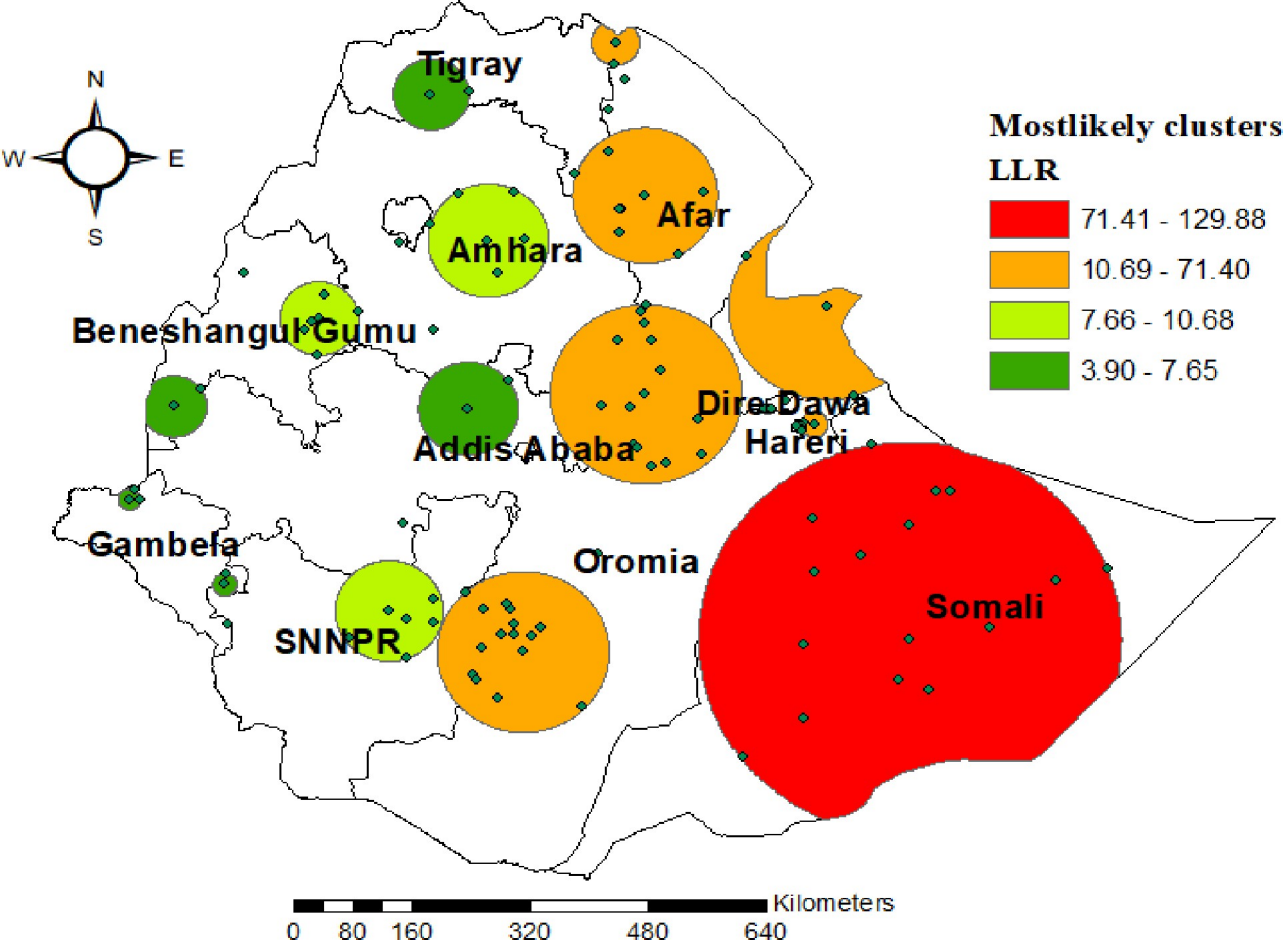
Spatial clustering of areas with high rate of home delivery among women who had live birth in the last five years preceding 2019 EDHS.

The SaTScan spatial analysis detected fourteen groups of statistically significant SaTScan clusters with high home rate of delivery. This implies that the prevalence of home delivery was higher inside the SaTScan circular window compared to outside the SaTScan window. The most likely primary SaTScan cluster of high home rate of delivery was detected (LLR = 129.9, p<0.000 in Somali region. The first secondary most likely spatial SaTScan cluster in the Afar region, part of SNNPR, Harari region and some part of (Oromia and Amhara region) (LLR = 71.4, p <0.000), (for detail **see Fig 4**).

The primary cluster centered at (6.505335 N, 43.486778 E) with 285 Km radius, RR of 1.79, and LLR of 129.9 at p<000 showed that women within the area had a 79% higher risk to give birth at home than women outside the area. The first secondary clusters were centered at (9.720204 N, 40.270550 E) with 129.6 km radius, RR of 1.66, and LLR of 71.4, p < 0.000 showed that women within the area had a 66% higher risk to give birth at home than women outside the area (For detail see Table 2).

**Table 2:** Show that the most likely clusters from a purely spatial scan statistic (Bernoulli model) of high rate of home delivery among 15–49 age women who had live births in the last five years preceding EDHS 2019 survey.

Most likely Clusters	Locational ID (clusters)	No. of clusters	No. of population	No. of case	Coordinates /	Radius (km)	Relative risk	LLR	P-Value
Primary Cluster	123, 138, 137, 135, 145, 136, 134, 131, 142, 140, 122, 133, 132, 141, 124, 129	16	423	368	(6.505335 N, 43.486778 E)	285	1.79	129.9	0.000
1^st^ secondary Cluster	40, 42, 43, 69, 41, 50, 28, 127, 68, 49, 88, 105, 106, 48, 47	15	332	272	(9.720204 N, 40.270550 E)	129.6	1.66	71.4	0.000
2^nd^ secondary cluster	113, 183, 186, 182, 181, 117, 115, 185, 187, 172, 89, 188, 184, 114,	14	340	266	(6.362562 N, 38.759281 E)	116.42	1.57	55.5	0.000
3^rd^ secondary cluster	39, 35	2	59	58	(14.300432 N, 39.911829 E)	32.54	1.93	34.56	0.000
4^th^ secondary Cluster	45, 29, 46, 44, 19, 34, 18, 33	8	226	172	(12.309340 N, 40.271524 E)	98.07	1.51	30.35	0.000
5^th^ secondary cluster	126, 32, 128	3	72	66	(10.832120 N, 42.520126 E)	133.3	1.8	27.94	0.000
6^th^ secondary cluster	107, 254, 255, 249, 248, 250	6	162	128	(9.312848 N, 42.343386 E)	18.39	1.56	27.26	0.000
7^th^ secondary cluster	103	1	22	21	(7.648661 N, 39.688764 E)	0	1.86	10.67	0.005
8^th^ secondary cluster	58, 60, 61, 83, 78, 57	6	129	91	(11.722588 N, 38.322763 E)	81.75	1.39	10.4	0.01
9^th^ secondary cluster	301	1	15	15	(9.614227 N, 41.997375 E)	0	1.95	10.01	0.012
10^th^ secondary cluster	304, 305, 303	3	97	71	(9.514266 N, 41.770584 E)	7.04	1.44	9.82.	0.014
11^th^ secondary cluster	38	1	27	24	(13.811395 N, 40.034385 E)	0	1.74	8.77	0.03
12^th^ secondary clusters	204, 191, 190, 189, 196, 198	6	104	74	(6.892369 N, 37.116362 E)	72.91	1.4	8.6	0.038
13^th^ secondary cluster	163, 148, 166, 162, 164, 80	6	141	96	(10.696075 N, 36.231791 E)	53.20	1.34	8.3	0.044

### Result of Multilevel logistic regression analysis

Table 3 shows results of the random intercept and random slope multilevel model. Model I (empty model) is with no covariates and model II is with individual level covariates included. Model III is with community level covariates and the last Model IV is the full model that included all level one and level two variables that were significant in model II and model III.

**Table 3:** Multilevel binary logistic regression analysis of individual and community-level factors among women who had live births within five years preceding (EDHS) 2019, Ethiopia.

Individual level variables	Null Model	Model II AOR (95%CI)	Model III AOR (95% CI)	Model IV AOR (95% CI)
Respondent age				
15–24 years	-	1.03(0.73, 1.46)	-	
25–34 years	-	0.82 (0.65, 1.02)	-	
35–49 years	-	1	-	
**Marital status**				
Never married		0.76(0.26, 2.22)		
Married		1.83(0.96, 3.47)		
Widowed		1.7(0.58, 4.94)		
Divorced		1		
Birth interval				
Less than 18 months	-	1	-	1
18 to 33 months	-	1.8(1.17, 2.84)*	-	1.8(1.19, 2.92)*
Above 33 months	-	2.095(1.36, 3.2)*	-	2.02(1.3, 3.12)*
**Antenatal care follow up**				
Less than four	-	1	-	1
4 and above		3.16(2.56, 3.89)**		2.72(2.2, 3.34)**
**Wealth index**				
Poorer	-	1	-	1
Middle	-	1.32(1.0, 1.73)	-	1.24(0.94, 1.63)
Rich	-	2.85(2.12, 3.84)**	-	2.2(1.62, 2.99)**
**Women’s educational level**				
No education	-	1	-	1
Primary	-	1.93(1.53, 2.44)**	-	1.8(1.44, 2.26)**
secondary	-	4.06 (2.41, 6.83)**	-	3.65(2.19, 6.1)**
Higher	-	4.42(1.67, 11.7)*	-	2.74(1.02, 7.34)*
**Media exposure**				
Not exposed at all	-	1	-	1
Exposed to either Radio/TV	-	1.24 (0.96, 1.59)	-	1.26(0.068, 1.62)
Exposed to both Radio and TV	-	5.5 (3.0, 10.06) **	-	4.6(2.52, 8.45)**
**Community Characteristics**				
**Region**				
Tigray	-	-	1.13(0.41. 3.08)	0.2(.045, 0.96)*
Afar	-	-	0.29(0.11, 0.82)*	0.125(026, 0.58)**
Amhara	-	-	0.77 (0.29, 2.02)	0.13(.046, 0.93)*
Oromia	-	-	0.40 (0.16, 1.03)	0.11(0.26, 0.509)*
Somalia	-	-	0.26(0.09, 0.74)*	0.18 (0.34, 0.87)*
Benishangul	-	-	1.72(0.63, 4.74)	0.47(0.10, 2.19)
SNNPR	-	-	0.68 (0.26, 1.79)	0.20 (0.046, 0.92) *
Gambela	-	-	0.91 (0.33, 2.49)	0.28 (0.61, 1.313)
Harari	-	-	0.91(0.33, 2.47)	0.37 (0.077, 1.73)
Addis Ababa	-	-	1	1
Dire Dawa	-	-	0.77(0.28, 2.10)	1.08(0.054, 1.216)
**Residence**				
Urban	-	-	1	
Rural	-	-	0.70(0.45, 1.09)	
**Community poverty status**				
High	-	-	1	1
Low	-	-	2.01(1.38, 2.93)*	0.88(0.597, 1.304)
**Community-women educational level**				
Low	-	-	1	1
High	-	-	2.44(1.64, 3.63)*	1.05(0.85, 1.83)
**Community ANC utilization**				
Low	-	-	1	1
High	-	-	6.1(4.18, 8.86)*	4.64(3.22, 6.7)**

Notes: * p < 0.05;

** p < 0.01, 1 = Reference category

Results in empty model (Model I) showed that there is a considerable level of variation in the odds of institutional delivery between communities (ICC = 57% which implies that 57% of the total variance in the institutional delivery was attributed to differences between communities.

In Model II only individual level variables were added. The results showed that women education level, household wealth index, birth interval, number of antenatal care visit, age of the women, iron brought during pregnancy, time of first antenatal visit, media exposure and marital status were significantly associated with birth at health institutions.

In Model III only community level variables were added to assess how much the variation in institutional delivery is explained by community variation. The result revealed that women’s residence, region, women residing in communities with low poverty level, residing in communities with low educational level and women residing in communities with low rate of antenatal care utilization were significantly associated with institutional delivery.

The final model (Model IV) included both the individual and community level characteristics simultaneously and the final result of model IV by part as follows:

Individual level effects: Women educational level, wealth index, media exposure, birth interval and antenatal care visit were significantly affects delivery in health facility.

The education of women increases the odd of giving birth at heath institution. Women who had primary education were 80% times (OR = 1.8: 95% CI: 1.44–2.26) more likely give birth at health institution as compared to those who had no education. Women who had secondary education were 3.65 times (OR = 3.65: 95% CI: 2.19–6.1) more likely to give birth at health institutions as compared to women who had no education and women who had diploma and higher were 2.74 times (OR = 2.74: 95% CI: 1.02–7.34) more likely to give birth at health institutions as compared to women who had no education. It is less likely that those women who didn’t received full antenatal care service during pregnancy to give birth at health institution as compared to those who had full antenatal care visit during pregnancy. That is those who had four and above ANC 2.27 times (AOR = 2.72, 95% CI:2.2, 3.34) more likely to give birth in health facility as compared to those who had less than four ANC visit during pregnancy. The combined wealth index status of household was positively associated with women’s health facility delivery. The odds of health facility delivery were 2.22 times (OR = 2.22; 95% CI: 1.62–2.99) more likely among women’s who were rich wealth index status as compared with women’s who were poor wealth index.

Those women who had both Radio and Television were 4.6 times (OR = 4.6; 95% CI: 2.52, 8.45) more likely to have health facility delivery as compared to those who hadn’t both of radio and television. The odds of health facility delivery were 80% times (OR = 1.8; 95% CI: 1.19, 2.92) more likely among women who space birth for 18 to 33 months and 2.02 times (OR = 2.02; 95% CI: 1.3, 3.12) more likely among women who space birth for more than 33 months as compared to those women who had birth interval less than 18 months.

### Community level effects

The study included 305 clusters, in which all the women who had live birth in the past five year before preceding survey.

Women living in communities with a high proportion of antenatal uptake were 4.64 times (OR = 4.64; 95% CI: 3.22, 6.7) to give birth at health facility than women living in communities with low proportion of antenatal care visit.

Those women who live in the Afar region were 87.5% times (AOR = 0.125;95% CI: 026, 0.58), in the Oromia region 89% times (AOR = 0.11; 95% CI: 0.26, 0.509), in the Somali region 82% times (AOR = 0.18; 95% CI: 0.34, 0.87), in the Tigray region 20% times (AOR = 0.2; 95% CI: 045, 0.96), in the Amhara region 87% times (AOR = 0.13; 95% CI: 046, 0.93) in SNNPR 80% times (AOR = 0.20; 95% CI: 0.046, 0.92). less likely to give birth at health facility as compared to children who were reside in capital city (Addis Ababa).

### Measures of variation (random-effects) and model fit statistics

As the multilevel logistic regression analysis results described in Table 4, Model I revealed statistically significant variation in institutional delivery across communities. More than half of (57%) of variation in the odds of institutional delivery is attributed to community-level factors (ICC = 57%).

**Table 4:** Measures of variation (random intercept models) and model fit statistics in institutional delivery among women who had live birth in the last five years preceding 2019 EM DHS.

Random effect result	Null model	Model II	Model III	Model IV
ICC(%)	57%	35.3%	30%	25.7%
PCV(%)	Reference	59%	66.4%	74%
MOR	7.4	3.6	3.17	2.76
Model fit statistics				
Log-likelihood	-2897.981	-1527.508	-2766.272	-1479.501
AIC	5799.962	3089.016	5564.544	3009.003
DIC	5795.962	3055.061	5532.544	2959

**Note** increased risk (in median) that one would have if moving to a neighborhood/cluster with a higher risk

After adjusting the model for individual-level factors (Model II), about 59% of the variation in the odds of institutional delivery was attributed to the individual level factors (PCV = 57%), and 35.3% of the variance in institutional delivery was attributed to community-level factors (ICC = 35.3%) (Table 4). Model III, which was adjusted for community-level factors, revealed that the community-level factors explained 66.4% of the variability in the odds of institutional delivery (PCV = 66.4%), and 30% of the variation among the clusters was attributed to community-level factors (ICC = 30%) (Table 4). The final best-fit model (model IV) was adjusted for both individual and community-level factors simultaneously. In this final model, about 25.7% of the variability among communities in the odds of institutional delivery was due to the community-level factors (ICC = 25.7%) and 74% of the variance in the odds of institutional delivery (PCV = 74%) across communities was attributed to both individual and community-level factors (Table 4). Including both individual and community level factors reduced the unexplained heterogeneity in institutional delivery between communities from MOR of 7.4 in the null model to the MOR of 2.76 in the final model, which equals 4.64. This showed that the likelihood of having an institutional delivery increased by 4.64 times when women moved from low to high-risk neighborhoods (Table 4).

### Model fit statistics

As shown in Table 4 below, a small number of AIC, DIC, and a large number of LLR in Model IV, indicating that the explanatory value of the model increases for Model IV. In other words, Model IV explained the determinants better than Model II and III; this makes the final model the best-fitted model than others.

## Discussion

According to the discoveries of this study, women’s education has a significant impact on institutional delivery. Higher levels of education increase the likelihood of giving birth in a health facility. The common explanations for this positive association include the fact that increased education improves women’s knowledge of health problems, raises awareness of the availability and accessibility of health services, increases female decision-making power, and causes changes in household dynamics. As a result, improving mothers’ educational and literacy status may be critical to strong institutional delivery.

According to the findings of this study, women who had four or more antenatal visits had high Oddes to give birth at a health care facility. Previous literature [30–34] found that attending ANC increases the likelihood of institutional delivery. This could be because antenatal care is a gateway to using delivery services. Health care providers may be able to encourage women to deliver in a health facility by emphasizing the importance of safe delivery in lowering maternal and early neonatal mortality [35].

Women who live in areas with a high rate of antenatal care uptake were also reported to have higher chance of giving a child in a hospital than women living in communities with a low rate of antenatal care uptake, a finding supported by several studies [30, 31]. High antenatal care utilization in the community reflects the community’s familiarity with women’s care and the health service use habits of women in the community, which plays an important role in positively influencing other women’s health seeking behavior. Furthermore, the health professional’s health education, counseling, and treatment services provided throughout Antenatal visit can result in behavioral improvements and increased potential benefits of seeking institutional delivery services.

The cumulative household wealth index was revealed to be a considerable predictor of health delivery. Women from middle to upper families were more likely than women from lower-income families to give birth in a health facility. This finding was consistent with previous research revealed in Nepal [36], east Africa [37], sub- Saharan Africa [38], India [39]. Perhaps in some African countries where maternal health care is provided for free [40], extra costs such as transportation and other potential costs for mothers and newborns to prevent poor health outcomes [41]. These could be due to higher socioeconomic status, which may enhance healthcare-seeking behavior and autonomy in healthcare decision-making because they can afford the necessary medical and transportation costs [42]. Whereas maternity and ambulance companies are free in Ethiopia, it is well noticeable that drug and transportation facilities are still out of pocket costs, as many drugs are not available in health institution because there are only a relatively limited number of ambulances.

Mothers who had a lot of media exposure had a higher chance of giving birth in a hospital. Prior research in Nigeria [43] Bangladesh [44] India [45]. The possible reason is that health information may improve health-seeking habits via various electronic and print media, as information about what services are available, where and when to get them, as well as the benefits and risks of accessing specific services, can be communicated via such media [46]

A birth interval of more than two years was discovered to be a significant factor in giving birth at a health facility. Numerous worldwide studies have found that an birth pacing of two years or more enhances maternal and child health, including the use of skilled care [47] This could be because mothers have more free time to rest and prepare themselves when they have sufficient time between pregnancies and use the facilities.

The spatial and multilevel analysis both realized that the geographic variation of institutional delivery throughout the country varied significantly. Significant hot—spot locations with a small number of patients with institutional delivery (high home delivery) were identified in the Afar, Amhara, southern Oromia, SNNPR, and most parts of Somalia. One explanation could be the discrepancy in the provision of maternal health services, as well as the poor accessibility of infrastructure such as roads for transportation in those regions’ border regions [48]. Furthermore, these community were more pastoral areas; as a consequence, relative to the rest, health facilities are not available [49]. This finding shows that public health planners and programmers should develop successful public health actions to improve institutional delivery in these substantial hot—spot areas with low institutional delivery.

And those mothers reside in capital city (Addis Ababa) and Dire-Dawa had higher odds of institutional delivery as compared to other parts of Ethiopian region. The consistent result has been reported in Ethiopia [50, 51]. The potential reason might be due to health facilities are easily nearby and highly aggregated in Addis Ababa and Dire-Dawa. on the other hand women in pastoral regions have poor access to education and are not permanent residents and because of these in these areas, there is limited availability and accessibility of maternal health services such as institutional delivery.

### Limitation

This study can’t determine causality because of the cross-sectional study design. Due to the irregular shape of the earth, a circular SatScan window may miss intervention areas.

### Strength

Regarding the strength of the methodology to consider the hierarchical nature of the EDHS data, a two-level mixed-effects logistic regression was used to handle both the fixed effects of individual and community factors and random effects to explain the between-cluster variations simultaneously. Furthermore, it is noted that EDHS data often collects individual data; it doesn’t collect data that describe the clusters directly except region and place of residence. As a result, this study endeavored to generate variables that can characterize communities by aggregating individual data into cluster values. This enabled the study to test whether community-level factors could influence the institutional delivery, in addition to individual-level factors. The other strength of this study was with estimation adjustments for representativeness of EDHS data, such as applying weighting of data considering sample designs during analysis of cross-tabulation and estimation to be representative of the Ethiopian population.

## Conclusion

This study observed a clustered pattern of areas with high home delivery in Ethiopia. Statistically significant clusters of high home birth areas were detected in the country’s Somali, Afar, SNNPR, and Oromiya regions. The individual-level characteristics (women’s educational level, having ANC follow up, birth spacing, rich wealth index and media exposure. Community-level characteristics geographic region, community ANC coverage were statistically significant factors of institutional delivery. Therefore, it is good if the federal ministry of health and other concerned maternal and child health programmers give priority to the areas with high home birth coverage identified in this study. It is also better to consider the individual, and community level determinant factors that may help planners, policy, and decision-makers emphasize individuals and communities.

## Abbreviations

ANC: Antenatal car
EA: Enumeration Area
EDHS: Ethiopian Demographic and Health Survey
GIS: Geographic Information Systems
HSTP: Health Sector Transformation Plan
ICC: Intra- Class Correlation
LISA: Local Indicator of Spatial Association
MOR: Median Odds Ratio
PCV: Proportional Change in Variance
SSA: sub-Sahara Africa
SDG: Sustainable Development Goal
USAID: United States Agency for International Development
